# Monthly Summary

**Published:** 1899-06

**Authors:** 


### Monthly Summary.

DR. BLACK'S THEORY.—Dr. Black says that all teeth are equally soft; that the dentine of all teeth are the same in density; that they have the same specific gravity; that a good tooth is no better than a bad tooth, that any filling material that will save one tooth will save any other, and that there is no choice except on the part of the dentist between lead or tin or gold or amalgam. Well, now I do not believe that bad teeth are always bad. I do not believe that teeth that are bad in the beginning are bad all through life. I am honest in being mistaken, if I am mistaken, but I believe I have seen changes in the teeth of some of my patients. I call to mind a patient who for ten years suffered from caries of the teeth. He was then leading a sedentary life. He changed his occupation, becoming a railroad man, thus securing plenty of air and exercise. His teeth required constant care. Now he will go sometimes for five or six years without a single cavity in his teeth. He is an unprofitable patient because his teeth do not require any care. Dr. Black would say that they could not change for the better.—Dr. Erwin T. Darby, in *International.*

---

DENTIFRICE SOAP.—Thymol, 25; extract of rhatany, 100; warm glycerin, 600; calcined magnesium, 50; borax 400; oil of peppermint 100; medicinal soap, q. s. to produce 3,000. Dissolve the thymol and extract of rhatany in the warm glycerin, and add the other ingredients, mixing thoroughly.—Form of *Bull. Gen. de Ther.*

SUPRAORBITAL NEURALGIA.—Recently a patient who was suffering from supraorbital neuralgia accompanied by intense injection of the conjunctiva was referred to me by an oculist for examination of the teeth. There was no dead pulps on that side of the mouth. No cavities in the teeth which were not already filled—most of the fillings being small. There was one large amalgam filling in the second superior molar which was removed, but the pulp was not exposed. From repeated tapping on this tooth, the injection of hot and then cold water, I decided that the responses were sufficient to justify the destruction of the pulp. This was done with arsenic and after forty-eight hours the pulp was removed, the lingual root being found calcified one-third of its length from the apex of the root. That night the patient suffered from a retinal hemorrhage, from which he recovered in about two weeks. The neuralgia ceased and the injection of the conjunctiva disappeared within two weeks and has not returned. Was the calcification of the pulp the cause of the neuralgia?—*Dental Review.*

---

THE INSERTION OF DENTURES.—I do not think the dentist is justified in allowing his patient to remain for even a period of three months after furnishing his mouth without furnishing it again; he loses to a great extent the original facial expression, and it takes a much longer time to become accustomed to the feel of denture than if it were inserted as quickly after the extraction of the teeth as possible, setting aside the inconvenience and impairment to health through being deprived of his dental armature.—H. Rose, "Brit. Jour."

---

TO CLEAN AN OIL-STONE.—Smear a flat block of wood with glycerine and fine pumice and rub the stone, face down, till all traces of previous usage have disappeared. This will greatly improve the working qualities of the stone. To *ruin* an oil-stone, clean it with kerosene.—*Odontographic Journal.*

DOES LICENSE TO PRACTICE MEDICINE PERMIT THE PRACTICE OF ORAL SURGERY.—A curious case for medico-legal decision has arisen in the State of Rhode Island, where registration for medical practice is granted upon application to the State Board of Health by graduates of recognized medical colleges, while dentists are required by a recent enactment to pass an examination before the State Board of Registration in Dentistry. A graduate in dental surgery, and in medicine, after having been registered by the State Board of Health, and securing his license, engaged in the practice of medicine, including dental and oral surgery. He was forthwith arrested for infraction of the law requiring examination and license for the practice of dental surgery, and the case now awaits judicial decision. Logically it would be reasoned that one qualified to practice medicine and so authorized to do by State authority, would at the same time be conceded the qualification and the license to practice any part of medicine, ophthalmology, otology, obstetrics, gynecology, oral and dental surgery, etc. To ask a qualified and licensed medical man to pass a special examination in oral and dental surgery, would not be more ridiculous than to ask him to pass a special examination and secure a special license for the practice of midwifery. It would seem, further, from the legal point of view, that when there is conflict in letter between existing legislation the spirit must be followed, and surely no one would contend that the part is greater than the whole. The question in Rhode Island seems a very simple one, and the solution should be attended with no difficulty or complication. It all revolves about the point whether an approved graduate in medicine shall be permitted to practice all branches of it.—"Dominion Dental Journal."

---

SOLDERING GOLD CROWNS.—Make a saturated solution of borax by boiling, until no more will dissolve. When the solder is wanted to flow, moisten with this solution, and it will run like a flash. Mix yellow ochre to a creamy consistency for painting the parts to be protected from solder.—J. T. Usher, *Cosmos*.

CINNAMON WATER AS A DRESSING.—The limited use of cinnamon water as a dressing for wounds caused me to give it an extended trial in my Bellevue clinic. The strength of the solution used was eight drops of the pure oil to a quart of water. This solution was used to syringe the parts and was applied to the dressing by the patients at home. In all minor surgical cases of recent wounds (tested in hundreds of cases) it can certainly be relied upon to take the place, fully, of that malodorous drug, iodoform. In burns of the first and second degree it never failed. The same with infected wounds. The dressings were changed every other day.

Case 1. Nellie G. received a severe lacerated wound of the wrist, severing both radial and ulnar arteries. A soiled towel was at once applied as a compress. After ligating the arteries, the wound was cleansed with the solution of cinnamon, and the patient instructed to keep the dressing constantly moistened with the same. The treatment extended from April 3 to June 15. Result, cured.

Case 2. Bridget K. severely burned on the right shoulder and one-third the distance to the elbow. The dates in this case are wanting. Writing from memory, the time consumed in this case was about three weeks. The patient pleaded very earnestly for a salve, but with much persuasion was induced to continue the treatment, with a most satisfactory result. Should any of your great army of readers have an untried agent which they wish tested, I will be pleased to use it for them and report. In conclusion I would say that I intend trying the oil of pepperment and gaultheria.—F. Soper, M. D., in "Dental Review."

---

TO MEND a plate temporarily, where a tooth or section has beee broken off leaving the pins in the rubber, wash the exposed surface thoroughly with soap and water and then with chloroform after drying thoroughly. Coat the surfaces with a mixture of equal parts of gutta-percha and resin, dissolved in chloroform lay a thin piece of gutta-percha over the broken surface of the plate, heat over a spirit lamp and press the tooth or section to place.—"Indiana Journal."

EPILEPSY.—The writer has never yet seen a case of so-called idiopathic epilepsy in which the kitchen did not figure. Of course there are other causes, but indigestion and malassimilation are the roots of evil in the great bulk of cases. It is remarkable how a series of seizures is cut short by the thorough cleaning of the alimentary tract.

In over a hundred cases taken at haphazard, not one was found in normal nutritive condition; forty per cent. of the stomachs were diluted, ninety per cent. of the alimentary tracts were more or less catarrhal.

Those who have dealt with epileptics will recall how frequently hemorrhoids and prolapse of the rectum are met with; and how prone they are to scorbutic taint. Almost without exception epileptics have weak, irritable, rapid hearts, showing simply a lowered vitality.—"Surgical and Dental News."

---

TOBACCO chewers' teeth wear away on the grinding surface rapidly, caused by the gritty substance naturally entering into the tobacco. The gums recede and are red and congested, and underneath the gum a narrow line of dark tartar is nearly always present and particles may be found still further towards the apex of the tooth I am now convinced that tobacco chewing does cause decay of the teeth, especially at the gingival portion of the teeth in the locality in which the cud is held; this is due to the formation of acids produced by the sugar or licorice used to flavor the tobacco.—Dr. John G. Harper, in *Digest.*

---

THERE is one thing, if not more, that carbolic acid seems to do better than anything else and that is the destruction of an abscess sack. Other caustics may do very well, but carbolic acid is excellent. It is not time yet to discard this remedy.—*American Dental Weekly.*

SUBSTITUTE FOR RUBBER GLOVES.—We all know tha in warm weather rubber gloves cannot be worn with comfort while engaged in prosthetic work; to subserve the same purpose the following suggestions for an ointment is taken from the "Medical Brief": "In the warm days that are now before us, when a rubber glove cannot be worn with comfort while engaged in prosthetic work, an ointment of honey for the hands will subserve the same purpose. It holds the dirt in suspension and dissolves very quickly when immersed in water, leaving the hands soft and clean. Take clarified honey and rose water, of each one pint, listerine two ounces. Mix and bottle. For winter use, add two or three ounces of glycerin."—"Int. Dental Journal."

---

TO TREAT the root canals of posterior teeth when the walls are broken down so badly that the rubber dam can not be adjusted, proceed as follows: Prepare the tooth as for a permanent filling, fill the pulp chamber with SSW dressing seal, allowing the filling to project from the pulp chamber as far as occlusion will permit. Build a wall of amalgam around this and at a subsequent sitting adjust the rubber dam, remove the seal and proceed with treatment.—*Indiana Journal.*

---

GUM sections in prosthetic dentistry are like chromos in art, they are all made in the same mold. There is no possibility of choice in the manner of setting each tooth to give it an individuality, indicating the character of the man, giving a little twist here, a little turn there, some a little longer, some a little shorter, individualizing the later especially, securing the proper relations of each tooth to the lips and the face.—Dr. Freeman, in *Headlight.*

---

BRIDGE OR PLATE?—If you have a tooth likely to last several years, it is better to have a small crown or bridge than to use an artificial plate and run the risk of injuring several teeth.—Dr. Whittaker, in "Jour. Brit. Asso."

